# Supplementary material for: In utero adenine base editing corrects multi-organ pathology in a lethal lysosomal storage disease
Source: Nat Commun. 2021 Jul 13;12:4291. doi: 10.1038/s41467-021-24443-8 (PMC8277817; doi:10.1038/s41467-021-24443-8)
Supplement: Supplementary file 1 — Supplementary Information [file 41467_2021_24443_MOESM1_ESM.pdf]

In utero adenine base editing corrects multi-organ pathology in a lethal lysosomal storage disease

Supplementary Information

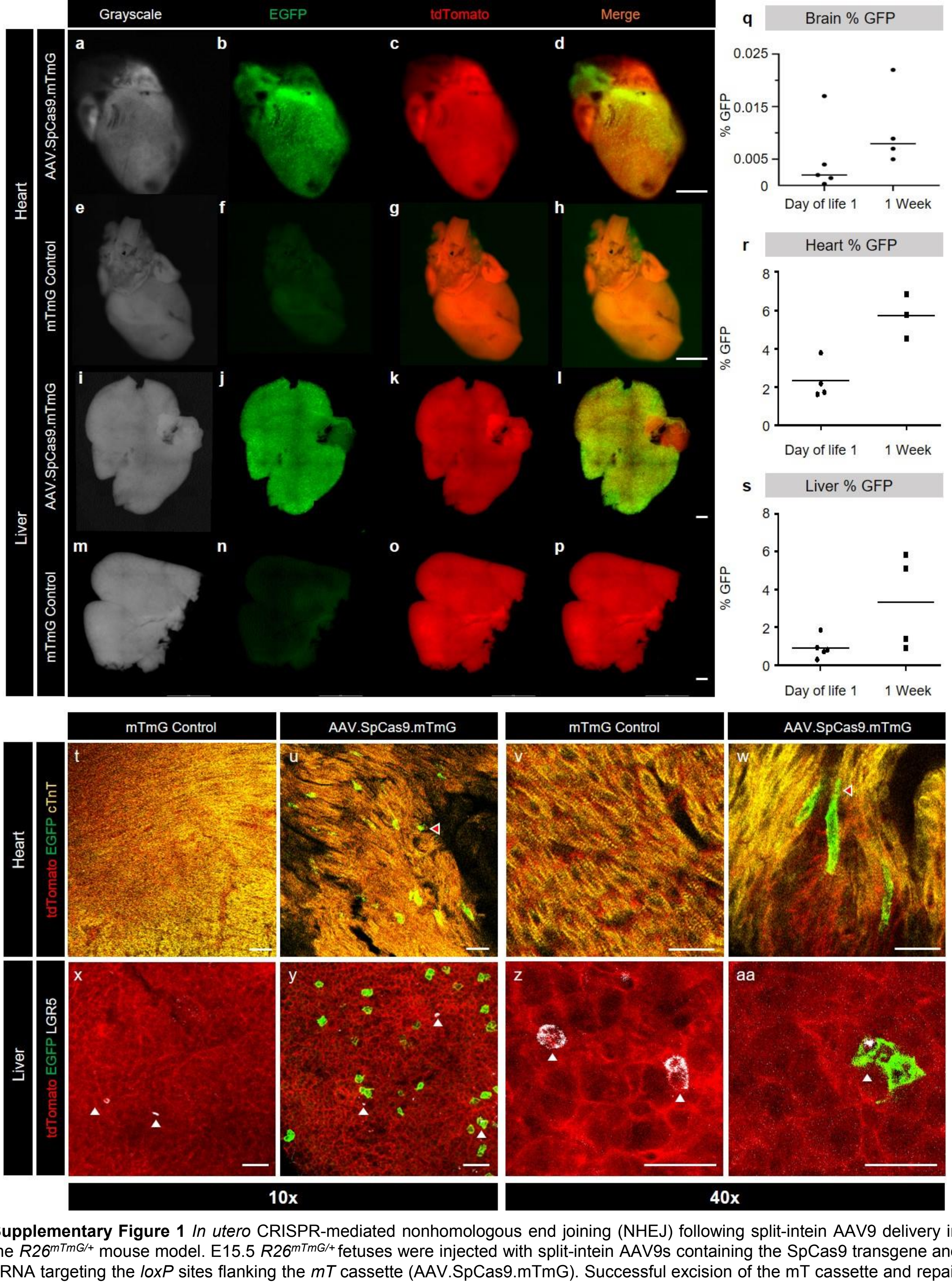

Supplementary Figure 1 *In utero* CRISPR-mediated nonhomologous end joining (NHEJ) following split-intein AAV9 delivery in the *R26*$^{mTmG/+}$ mouse model. E15.5 *R26*$^{mTmG/+}$ fetuses were injected with split-intein AAV9s containing the SpCas9 transgene and gRNA targeting the *loxP* sites flanking the *mT* cassette (AAV.SpCas9.mTmG). Successful excision of the mT cassette and repair via NHEJ results in expression of green fluorescence. **(a-p)** The hearts and livers of prenatally injected mice and uninjected *R26*$^{mTmG/+}$ controls were analysed at 1 week of age by stereomicroscopy (experimental heart, a-d; control heart, e-h; experimental liver, i-l; control liver, m-p) for GFP expression. (a-p) Scale bar=1mm. **(q-s)** Brains, hearts, and livers of day-of-life 1 mice (n=5 for brain and liver and n=4 for heart) and 1 week old (n = 4 for brain and liver and n=3 for heart) *R26*$^{mTmG/+}$ mice prenatally injected with AAV.SpCas9.mTmG were assessed by flow cytometry for GFP expression. **(t-aa)** Whole mount IHC of heart with staining for troponin (yellow, t-w) and the liver with staining for LGR5 (white, x-aa) in 1 week old *R26*$^{mTmG/+}$ mice prenatally injected with AAV.SpCas9.mTmG (u,w,y,aa) and uninjected *R26*$^{mTmG/+}$ mice (t,v,x,z). (t,u,x,y) Scale bar=50µm. Red-filled arrowheads identify cardiomyocytes. (v,w,z,aa) Scale bar=25µm. White arrowheads identify LGR5+ cells. EGFP, green; TdTomato, red; cTnT, cardiac troponin, yellow; LGR5, leucine-rich repeat-containing G-protein receptor 5, white. Source data are provided as a Source Data file.

a

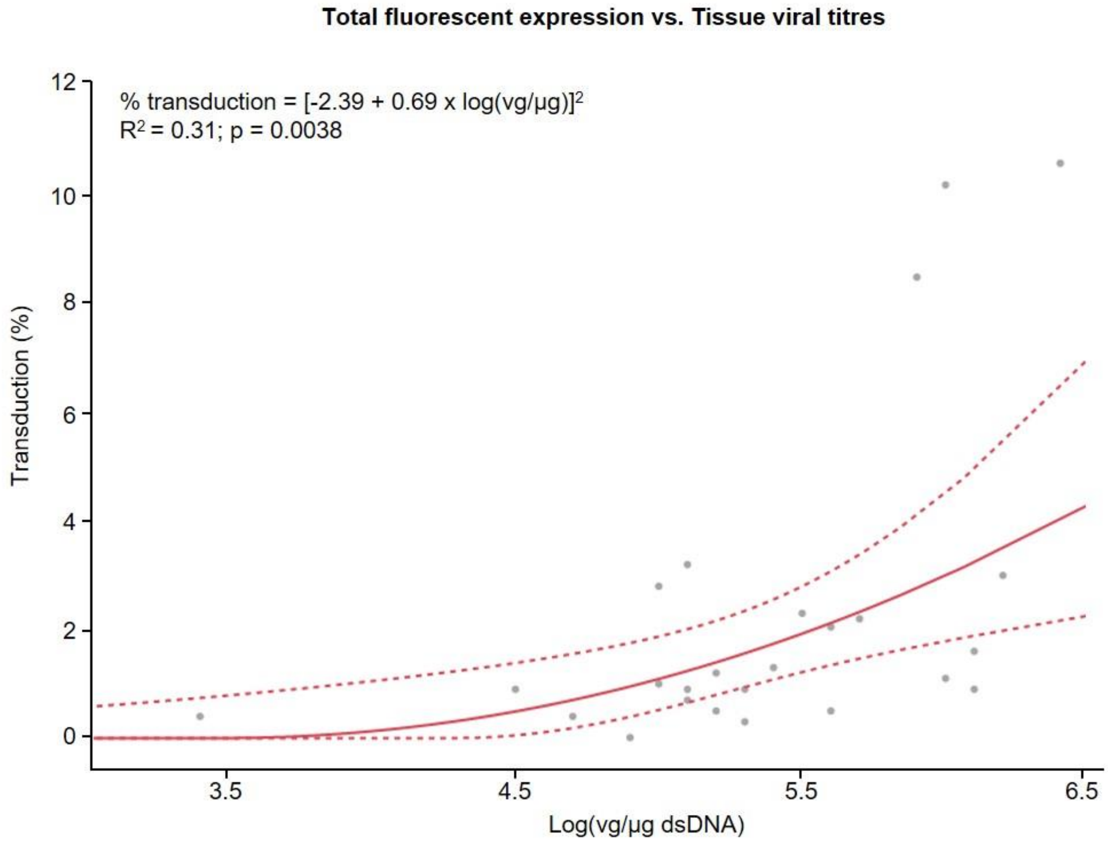

b

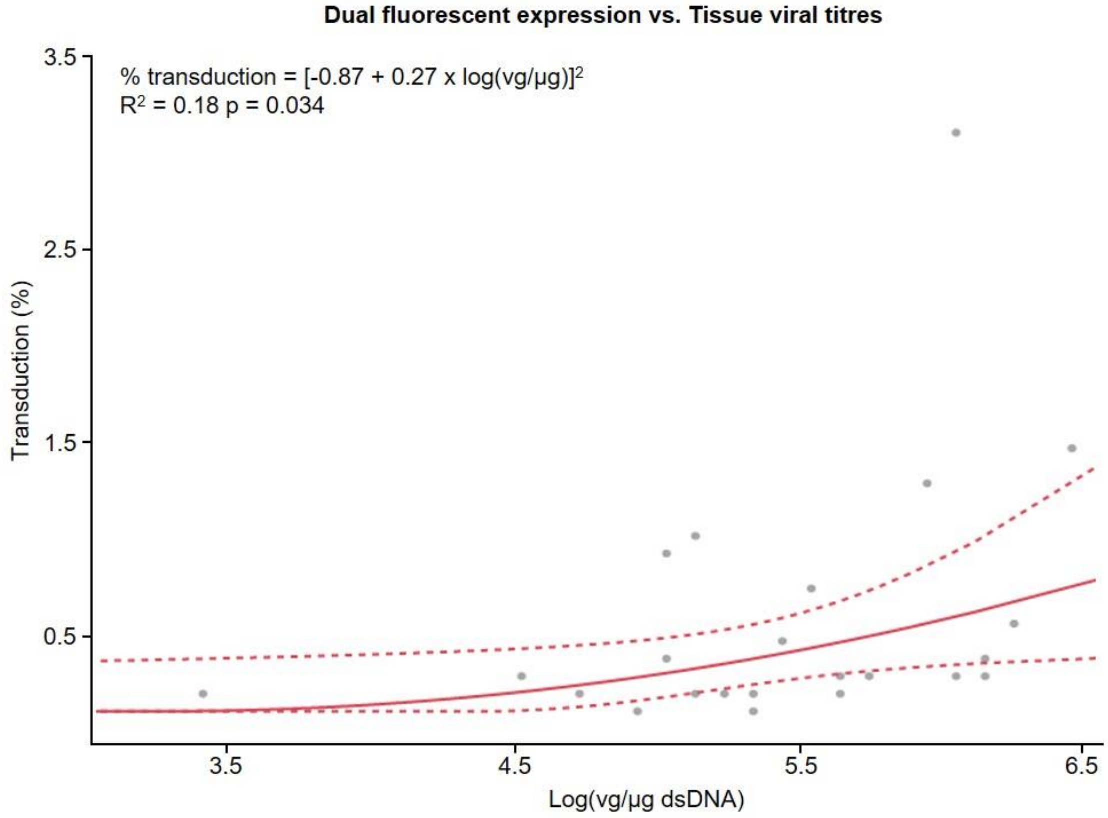

Supplementary Figure 2 The relationship between tissue viral titres and expression of fluorescent reporters following in utero dual AAV9 delivery in C57BL/6 mice. E15.5 fetuses were injected with a 1:1 ratio of AAV9 CMV-GFP and AAV9 CMV-mCherry. Brain, heart, and liver were harvested at 7 days post injection and evaluated using flow cytometry for total fluorescent expression (CD45-GFP+, CD45-mCherry+, and/or CD45-GFP+mCherry+) or dual fluorescent expression (CD45-GFP+mCherry+). Tissue AAV2 inverted terminal repeats indicating the presence of viral DNA were assessed by quantitative PCR. **(a, b)** Quadratic predictive equations describing the relationship between transduction and viral titres were generated for total fluorescent transduction (a) and dual AAV transduction (b). Y axes depict transduction percent as determined via flow cytometry and X axes depict $\log_{10}$(vector genome copies per µg double-stranded DNA). Dotted lines represent 95% prediction confidence intervals. The statistical significance of regression parameters was assessed at the α=0.05 level. Coefficients were assessed using a t-distribution with n-2 degrees of freedom. Source data are provided as a Source Data file.

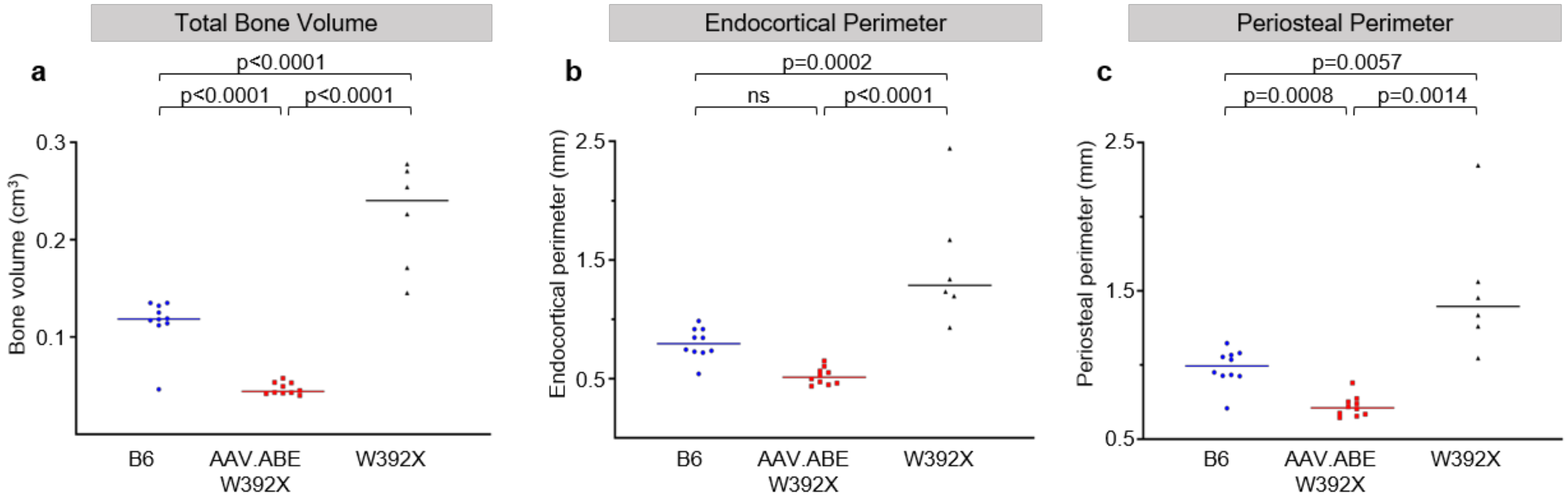

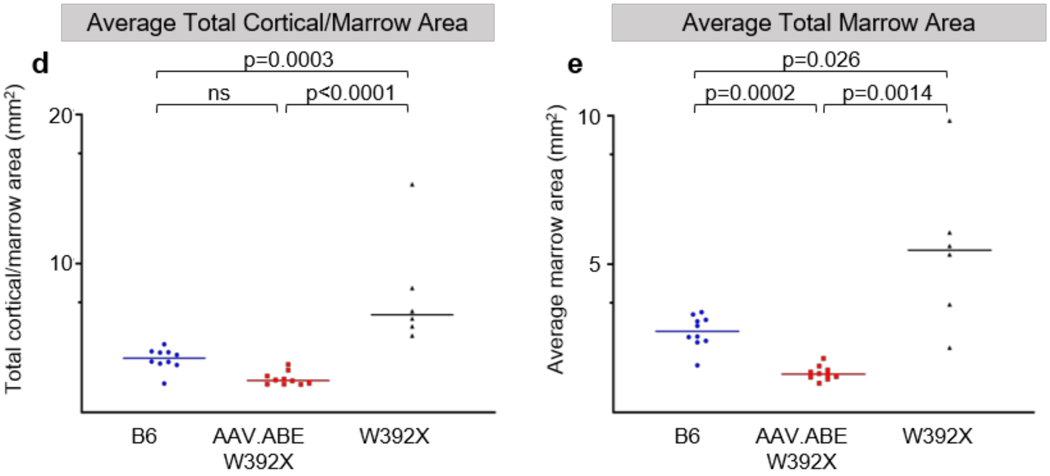

Supplementary Figure 3 In utero base editing improves the femoral bone phenotype in *IDUA-W392X* mice. **(a-e)** CT femur parameters were measured in 6-month-old B6 (N=10), 6-month-old *IDUA-W392X* mice prenatally injected with AAV.ABE.Idua (N=10), and surviving uninjected *IDUA-W392X* mice (N=6). Two-tailed Wilcoxon test for multiple comparisons was used to assess parameters given differing variances; α=0.05. CT, computed tomography scan. AAV.ABE indicates AAV.ABE.Idua. Source data are provided as a Source Data file.

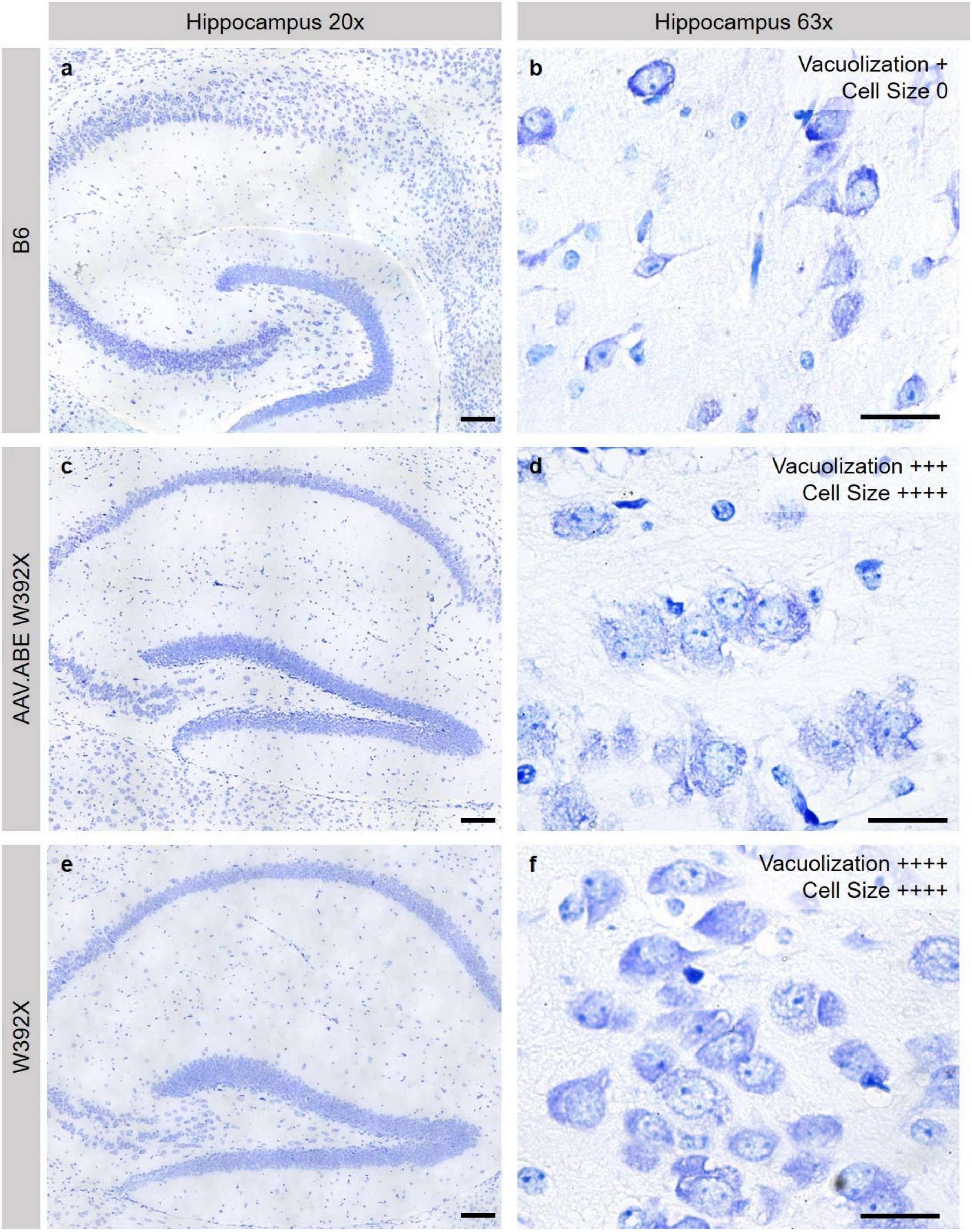

Supplementary Figure 4 Hippocampal cellular pathology following in utero base editing. **(a-f)** Representative histology with Toluidine blue staining of the hippocampal region in 6-month-old C57BL/6 mice (a, b), prenatal AAV.ABE.Idua injected *IDUA-W392X* mice (c, d), and uninjected *IDUA-W392X* mice (e, f). Blinded assessments of cellular vacuolization and cell size were conducted in prenatal AAV.ABE.Idua injected mice (n=10), C57BL/6 mice (n=2), and *IDUA-W392X* mice (n=2). Vacuolization scores were assessed as follows: 0, no cytoplasmic vacuoles; +, rare vacuolated cell (<1%); ++, cytoplasmic vacuoles in 0–10% of cells; +++, cytoplasmic vacuoles in 10–25% of cells; ++++, cytoplasmic vacuoles in >25% of cells. Cell size was assessed compared to C57BL/6 mice as follows: 0, equivalent; +, 0-25% larger; ++, 25-50% larger; +++, 50-75% larger; ++++, >75% larger. Mean pathologic scores within groups are displayed in inset panels. (a,c,e) Scale bar=100µm. (b,d,f) Scale bar=25µm.

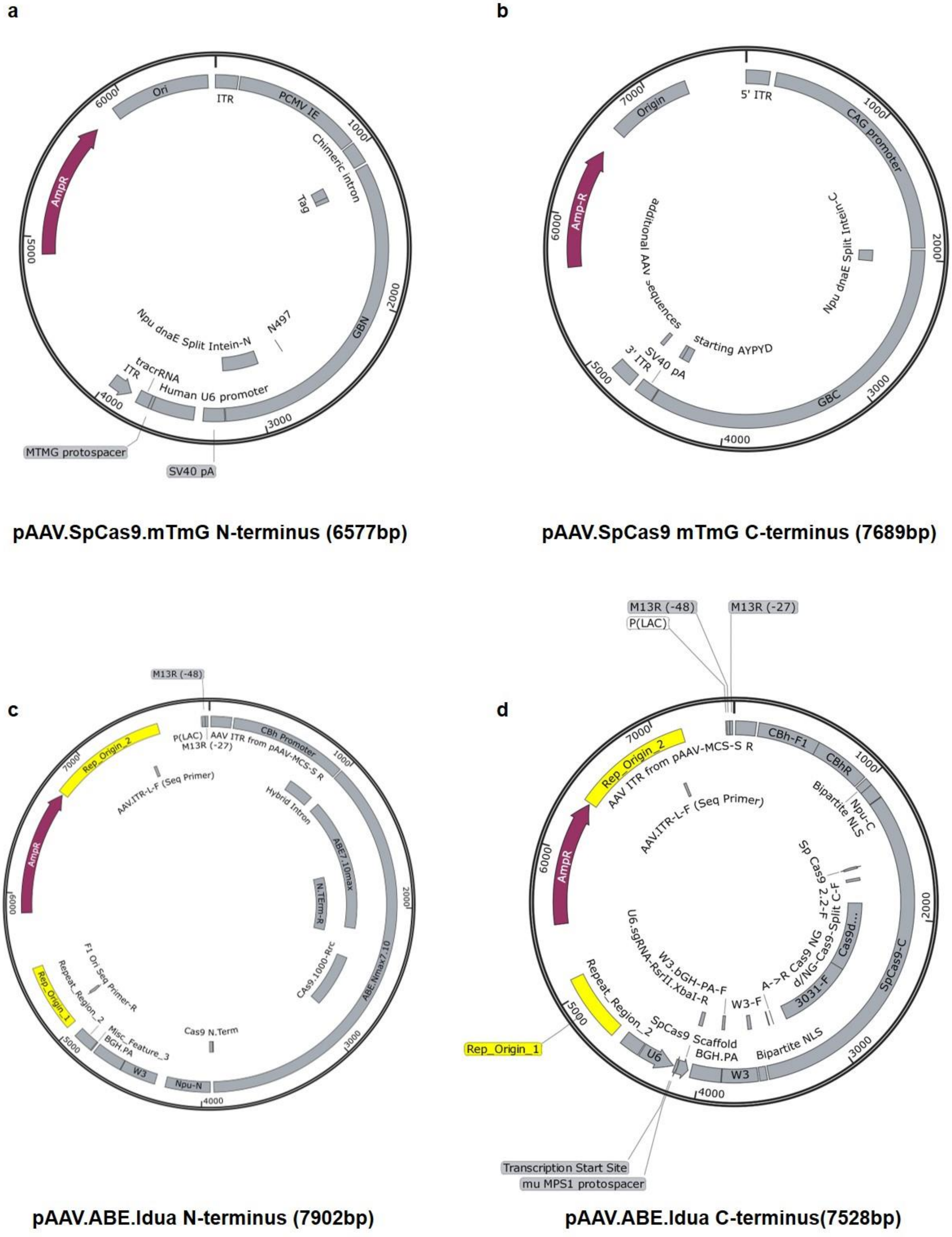

Supplementary Figure 5 Maps of plasmids used for AAV vector construction.

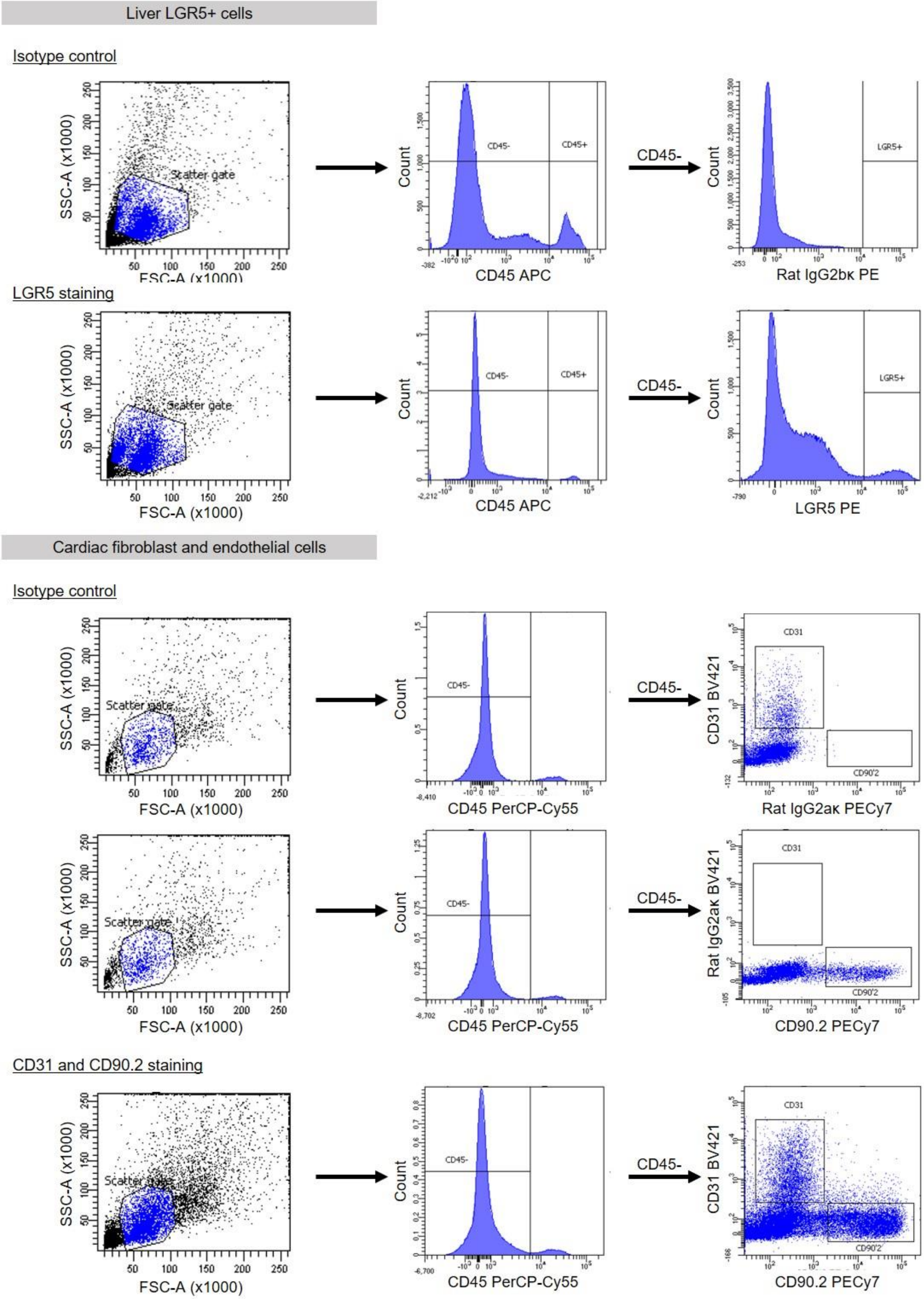

Supplementary Figure 6 Flow cytometry plots used for sorting CD45^{-}LGR5^{+} liver cells, CD45^{-}CD31^{-}CD90.2^{+} cardiac fibroblasts, and CD45^{-}CD31^{+}CD90.2^{-} cardiac endothelial cells as noted in Figures 1d-e.

Month	Group 1	Group 2	P-value
**1**	W392X	B6	<0.0001
	W392X	AAV.ABE prenatal	0.0119
	B6	AAV.ABE prenatal	0.0036
**2**	W392X	B6	<0.0001
	W392X	AAV.ABE prenatal	<0.0001
	B6	AAV.ABE prenatal	<0.0001
**3**	W392X	B6	<0.0001
	W392X	AAV.ABE prenatal	0.0434
	B6	AAV.ABE prenatal	<0.0001
**4**	W392X	B6	<0.0001
	B6	AAV.ABE postnatal	<0.0001
	W392X	AAV.ABE prenatal	0.0002
	B6	AAV.ABE prenatal	0.0006
	AAV.ABE prenatal	AAV.ABE postnatal	0.0215
	W392X	AAV.ABE postnatal	0.4661
**5**	W392X	B6	<0.0001
	B6	AAV.ABE postnatal	<0.0001
	W392X	AAV.ABE prenatal	0.00012
	B6	AAV.ABE prenatal	<0.0001
	AAV.ABE prenatal	AAV.ABE postnatal	0.3959
	W392X	AAV.ABE postnatal	0.0682
**6**	W392X	B6	0.0002
	W392X	AAV.ABE prenatal	0.0002
	B6	AAV.ABE prenatal	0.8146

Supplementary Table 1 Exact p-values for urinary gag comparisons as described in Figures 2a and 7b.

Dual AAV 2/9	Titre (GC/mL)
AAV.ABE.Idua C-terminus	$2.8x10^{12}$
AAV.ABE.Idua N-terminus	$5.8x10^{12}$
AAV.mTmG C-terminus	$1.5x10^{11}$
AAV.mTmG N-terminus	$1.2x10^{11}$
AAV.GFP	$1.0x10^{13}$
AAV.mCherry	$1.0x10^{13}$

Supplementary Table 2 Viral vector titres

Target	Forward Primer	Reverse Primer
**On-target**		
Idua	TGCTAGGTATGAGAGAGCCA	AGTGTAGATGAGGACTGTGGT
**Off-target**		
Intron:1700010I14Rik	GGGATTGCTCTGCTCTGTCT	TGTGTAAGAGTGGGCCATGT
Intron:Wnt11	CAGGCTTGAACACACACACA	AAAATCCCGTTGAGACCCCA
Intergenic:Papl-Fbxo27	CAACATTTGGAAGTCTGAGGC	TGCTGGGGTTACAAGGGTG
Intergenic:Fgf9-Gm25614	ACTGCAGGAATGGAAAACTCC	CTCTAGAGACCCTGTGCTGG
Intergenic:Gm12106-Stc2	AGGCCTTCGATCAGACATCA	CAACAACATGGCTGCTCAGG
Intergenic:Gm26190-B3gat1	CCTTCACTCTCTTGGGCCTT	CAGTGTCAGCAAAGGGAAGC
Intergenic:Ccdc85c-Hhipl1	ACAAGGAGGGGTGTGTGTAC	CTGCTGAGAGGTCCTGGAG
Intron:Osbpl1a	GCCCACTTAATAACCCTGTGT	GCAGGAGGGGTCATTGATCT
Intron:Blnk	ACAGCACTGAGAAGGGACAA	CGGGAGGGATCGTAAAGTGA
Intron:Rhoj	TTGGCTAGTCTCCGTGTGAA	GGGGTCTAGAGGTCTTTGGG
**qPCR**		
AAV2 ITR	GGAACCCCTAGTGATGGAGTT	CGGCCTCAGTGAGCGA

Supplementary Table 3 Primers used for Sanger sequencing, NGS in on- and off-target analysis, and quantitative PCR.

Protospacer and PAM	Location	CFD Off-target Score
ACTCTAGGCAGAGGTCTCAA AGG	Exon:Idua	
GTTCTAGACTGAGGTCTCAA GGG	Intron:1700010I14Rik	0.802139
ACTCCAAGCTGGGGTCTCAA CGG	Intron:Wnt11	0.637255
ACTCTAGGCTAGAGTCTCAA AGG	Intergenic:Papl-Fbxo27	0.588235
ACTTTTGACAGAGGTATCAA GGG	Intergenic:Fgf9-Gm25614	0.571429
ATTCCAGCCAGAGGTATCAA AGG	Intergenic:Gm12106-Stc2	0.559441
AGTTCAGACAGAGGTCTCAA AGG	Intergenic:Gm26190-B3gat1	0.556522
GCTCCAGGCAGAGGTCCCAG GGG	Intergenic:Ccdc85c-Hhipl1	0.539792
GAACTAAGCAGAGGTCTCAA AGG	Intron:Osbpl1a	0.519481
GCTCTGAGCAGAGGTCCCAA CGG	Intron:Blnk	0.504202
ACTCTACACAGAGGTACCAA TGG	Intron:Rhoj	0.485294

Supplementary Table 4 Off-target sites for *IDUA*

Type	Antibody	Catalog #	Clone #	(Host, Source)
IF	Anti-Cardiac Troponin I	PA5-28964	Polyclonal	(rabbit, Thermo Fisher Scientific)
IF	Anti-LGR5	LS C804326	Polyclonal	(rabbit, LSBio)
IF	Anti-IDUA C-terminus	AB178808	Polyclonal	(rabbit, Abcam)
IF	Anti-GFP	AB_2307313	Polyclonal	(chicken, Aves Labs)
IF	Alexa Fluor 647	AB150075	Polyclonal	(donkey, Abcam)
IF	Alexa Fluor 488	AB150153	Polyclonal	(donkey, Abcam)
IF	Alexa Fluor 514	A31558	Polyclonal	(goat, Invitrogen)
FC	Anti-CD45-PerCP-Cyanine5.5	45-04541-82	30-F11	(rat, eBioscience)
FC	Anti-CD31-Brilliant Violet 421	102424	390	(rat, Biolegend)
FC	Anti-CD90.2-PE-Cyanine7	25-0902-82	53-2.1	(rat, eBioscience)
FC	Anti-LGR5-PE	130-111-201	DA04-10E8.9	(rat, Miltenyi)
FC	Anti-CD45-APC	17-451-82	30-F11	(rat, eBioscience)
FC-Isotype	IgG2a Kappa PE Cyanine7	25-4321-82	eBR2a	(rat, eBioscience)
FC-Isotype	IgG2a Kappa Brilliant Violet 421	400535	RTK2758	(rat, Biolegend)
FC-Isotype	IgG2b Kappa PE	553989	A95-1	(rat, BD Pharmingen)
EL	Anti-SpCas9	A-9000-100	7A9	(mouse, Epigentek)

Supplementary Table 5 Antibodies used for immunofluorescence (IF), flow cytometry (FC), flow cytometry isotype control (FC-Isotype), and enzyme-linked immunosorbent assay (EL).
