# Supplementary material for: In utero adenine base editing corrects multi-organ pathology in a lethal lysosomal storage disease
Source: Nat Commun. 2021 Jul 13;12:4291. doi: 10.1038/s41467-021-24443-8 (PMC8277817; doi:10.1038/s41467-021-24443-8)
Supplement: Supplementary file 3 — Reporting Summary [file 41467_2021_24443_MOESM3_ESM.pdf]

nature research

Corresponding author(s): William Peranteau

Last updated by author(s): Apr 29, 2021

Reporting Summary

Nature Research wishes to improve the reproducibility of the work that we publish. This form provides structure for consistency and transparency in reporting. For further information on Nature Research policies, see our Editorial Policies and the Editorial Policy Checklist.

Statistics

For all statistical analyses, confirm that the following items are present in the figure legend, table legend, main text, or Methods section.

n/a	Confirmed	
☐	☒	The exact sample size (*n*) for each experimental group/condition, given as a discrete number and unit of measurement
☐	☒	A statement on whether measurements were taken from distinct samples or whether the same sample was measured repeatedly
☐	☒	The statistical test(s) used AND whether they are one- or two-sided<br>*Only common tests should be described solely by name; describe more complex techniques in the Methods section.*
☒	☐	A description of all covariates tested
☐	☒	A description of any assumptions or corrections, such as tests of normality and adjustment for multiple comparisons
☐	☒	A full description of the statistical parameters including central tendency (e.g. means) or other basic estimates (e.g. regression coefficient) AND variation (e.g. standard deviation) or associated estimates of uncertainty (e.g. confidence intervals)
☐	☒	For null hypothesis testing, the test statistic (e.g. *F*, *t*, *r*) with confidence intervals, effect sizes, degrees of freedom and *P* value noted<br>*Give P values as exact values whenever suitable.*
☒	☐	For Bayesian analysis, information on the choice of priors and Markov chain Monte Carlo settings
☒	☐	For hierarchical and complex designs, identification of the appropriate level for tests and full reporting of outcomes
☒	☐	Estimates of effect sizes (e.g. Cohen's *d*, Pearson's *r*), indicating how they were calculated

Our web collection on statistics for biologists contains articles on many of the points above.

Software and code

Policy information about availability of computer code

Data collection	microCT: Inveon Acquisition Workplace 1.5a
Data analysis	Off-target: CRISPOR (http://crispor.tefor.net); NGS: CRISPResso2 (https://crispresso.pinellolab.partners.org/); microCT: Dragonfly v4.1; Open Field: FFMPEG 4.2, MouBeAT 1 plugin for NIH ImageJ 1.53c; ELISA: SoftMax Pro 6.3; Statistics: JMP 14.3, GraphPad Prism version 8.0.0; TTE: FUJIFILM Vevo Lab v.3.2.0

For manuscripts utilizing custom algorithms or software that are central to the research but not yet described in published literature, software must be made available to editors and reviewers. We strongly encourage code deposition in a community repository (e.g. GitHub). See the Nature Research guidelines for submitting code & software for further information.

Data

Policy information about availability of data

All manuscripts must include a data availability statement. This statement should provide the following information, where applicable:

- Accession codes, unique identifiers, or web links for publicly available datasets
- A list of figures that have associated raw data
- A description of any restrictions on data availability

DNA sequencing data has been deposited on the NCBI Sequence Read Archive with the associated BioProject ID: PRJNA725910 and Accession numbers SAMN18915311-1895484. Figures 1-8 and Supplementary Figures 1-5 are associated with raw data that are provided as a Source Data file.

Field-specific reporting

Please select the one below that is the best fit for your research. If you are not sure, read the appropriate sections before making your selection.

[x] Life sciences [ ] Behavioural & social sciences [ ] Ecological, evolutionary & environmental sciences

For a reference copy of the document with all sections, see nature.com/documents/nr-reporting-summary-flat.pdf

Life sciences study design

All studies must disclose on these points even when the disclosure is negative.

Sample size

Based on published literature and initial experiments that determined the range of variation between diseased-control and wild-type animals, we estimated that the N used in this study would adequately power our intended statistical assessments. In particular, prior studies of MPS-IH in the mouse model demonstrated the ability to detect differences between treatment and experimental groups at N=5-10 per group (references below). In addition, based on a pilot experiment in two mice, we were able to detect evidence of liver gene editing of ~27%, heart gene editing of ~14%, and brain gene editing of ~1.3%. Using the minimum of these three statistics, we calculated requiring a minimum of 8 mice per group to detect an editing difference between experimental and control of 1.3% ± 0.9 (mean ± 1xSD) at α=0.05 and power of 80%. The sample size required for detecting editing in other organs was substantially lower given the greater magnitude of expected difference between experimental and control mice. Finally, power calculations were generated for other biochemical (IDUA and GAG assays) using the same assumptions. The average number of mice per group required was 6 and so we elected to inject 10 mice and use 10-14 control mice per comparison to maximize precision.

Hartung, S. D., Frandsen, J. L., Pan, D., Koniar, B. L., Graupman, P., Gunther, R., ... & McIvor, R. S. (2004). Correction of metabolic, craniofacial, and neurologic abnormalities in MPS I mice treated at birth with adeno-associated virus vector transducing the human α-L-iduronidase gene. Molecular Therapy, 9(6), 866-875.

Kobayashi, H., Carbonaro, D., Pepper, K., Petersen, D., Ge, S., Jackson, H., ... & Kohn, D. B. (2005). Neonatal gene therapy of MPS I mice by intravenous injection of a lentiviral vector. Molecular Therapy, 11(5), 776-789.

Ou, Li, et al. "ZFN-mediated in vivo genome editing corrects murine hurler syndrome." Molecular Therapy 27.1 (2019): 178-187.

Data exclusions

No data were excluded from this study.

Replication

Analyses were performed in duplicate where appropriate and multiple litters of injected and control animals were combined for final analysis. All attempts at replication were successful.

Randomization

Based on the sex distribution of the injected mice, we randomly selected a similar composition of age-matched disease-control and wild-type animals.

Blinding

Where possible, phenotypic measurements were analysed by a blinded investigator. Echocardiography and open field tests were analyzed by a blinded investigator. Based on the author's knowledge of CT analysis and the need to develop an analytic pipeline, the interpretation of initial pilot CT scans was not blinded but subsequent experimental CT studies were. To achieve blinding, animals were assigned a unique identifier that was maintained in a spreadsheet. Assays were performed blind to the status of the animal (treatment vs. control) and results were coded with the unique identifier. At the end of analyses, codes were de-identified to assign treatment vs. control status for statistical analyses.

Reporting for specific materials, systems and methods

We require information from authors about some types of materials, experimental systems and methods used in many studies. Here, indicate whether each material, system or method listed is relevant to your study. If you are not sure if a list item applies to your research, read the appropriate section before selecting a response.

Materials & experimental systems

n/a	Involved in the study
[ ]	[x] Antibodies
[x]	[ ] Eukaryotic cell lines
[x]	[ ] Palaeontology and archaeology
[ ]	[x] Animals and other organisms
[x]	[ ] Human research participants
[x]	[ ] Clinical data
[x]	[ ] Dual use research of concern

Methods

n/a	Involved in the study
[x]	[ ] ChIP-seq
[ ]	[x] Flow cytometry
[x]	[ ] MRI-based neuroimaging

Antibodies

Antibodies used

Anti-Cardiac Troponin I PA5-28964 Polyclonal (rabbit, Thermo Fisher Scientific)
Anti-LGR5 LS C804326 Polyclonal (rabbit, LSBio)
Anti-IDUA C-terminus AB178808 Polyclonal (rabbit, Abcam)
Anti-GFP AB_2307313 Polyclonal (chicken, Aves Labs)
Alexa Fluor 647 AB150075 Polyclonal (donkey, Abcam)
Alexa Fluor 488 AB150153 Polyclonal (donkey, Abcam)
Alexa Fluor 514 A31558 Polyclonal (goat, Invitrogen)
Anti-CD45-PerCP-Cyanine5.5 45-04541-82 30-F11 (rat, eBioscience)
Anti-CD31-Brilliant Violet 421 102424 390 (rat, Biolegend)
Anti-CD90.2-PE-Cyanine7 25-0902-82 53-2.1 (rat, eBioscience)
Anti-LGR5-PE 130-111-201 DA04-10E8.9 (rat, Miltenyi)
Anti-CD45-APC 17-451-82 30-F11 (rat, eBioscience)
Isotype IgG2a Kappa PE Cyanine7 25-4321-82 eBR2a (rat, eBioscience)
Isotype IgG2a Kappa Brilliant Violet 421 400535 RTK2758 (rat, Biolegend)
Isotype IgG2b Kappa PE 553989 A95-1 (rat, BD Pharmingen)

Validation

All antibodies were purchased from commercial vendors who have previously validated the specificity of each antibody. Additional validation was performed as described below.

Immunofluorescence antibodies were validated using serial dilution with disease-control and wild-type samples. Validation was performed by initially staining positive and negative control specimens at three dilutions to identify the appropriate staining patterns prior to staining experimental specimens. References for utilized primary antibodies are as follows:

Anti-GFP AB_2307313 polyclonal (chicken, Aves Labs)
MoChenaTakakoKatobYukioKato (2019), 'Data on localization of coxsackievirus and adenovirus receptor (CAR) in the embryonic rat brain.' Science Direct. 10.1016/j.dib.2019.103726.
Beatriz del Blanco, Deisy Guiretti, Romana Tomasoni, María T. Lopez-Cascales, Rafael Muñoz-Viana, Michal Lipinski, Marilyn Scandaglia, Yaiza Coca, Román Olivares, Luis M. Valor, Eloísa Herrera, Angel Barco (2019), 'CBP and SRF co-regulate dendritic growth and synaptic maturation.' Cell Death & Differentiation. 10.1038/s41418-019-0285-x.
Marina A. Silveira, Thais T. Zampieri, Isadora C. Furigo, Fernando Abdulkader, Jose Donato Jr., Renata Fraza~o (2019), 'Acute effects of somatomammotropin hormones on neuronal components of the hypothalamic-pituitary-gonadal axis.' Brain Research. 10.1016/j.brainres.2019.03.003.
Xuefeng Yuan, Ying Huang, Sarita Shah, Hua Wu, and Laurent Gautron (2016), 'Levels of Cocaine- and Amphetamine-Regulated Transcript in Vagal Afferents in the Mouse Are Unaltered in Response to Metabolic Challenges.' eNeuro. 10.1523/ENEURO.0174-16.2016.

Flow cytometric antibody validation was performed first by using previously published antibodies, second by testing each antibody on control mice prior to experimental mice, and third by performing isotype controls for each identifiable population.

References for utilized primary antibodies are as follows:

Cardiac subpopulations were stained using antibodies described by Prahl et al. (https://www.ahajournals.org/doi/abs/10.1161/res.121.suppl_1.478).

Anti-CD-45-PerCP-Cyanine5.5 45-04541-82 30-F11
McClendon, J., Jansing, N. L., Redente, E. F., Gandjeva, A., Ito, Y., Colgan, S. P., ... & Zemans, R. L. (2017). Hypoxia-inducible factor 1α signaling promotes repair of the alveolar epithelium after acute lung injury. The American journal of pathology, 187(8), 1772-1786.

Anti-CD31-Brilliant Violet 421 102424 390
Baldwin HS, Shen HM, Yan HC, et al. Platelet endothelial cell adhesion molecule-1 (PECAM-1/CD31): alternatively spliced, functionally distinct isoforms expressed during mammalian cardiovascular development. Development. 1994; 120(9):2539-2953. View reference

Anti-CD90.2-PE-Cyanine7 25-0902-82 53-2.1
Prahl, J. D., Weiland, M., Milliron, H., Kort, E. J., & Jovinge, S. (2017). Cell-type Specific Surface Markers Effectively Isolate Cardiac Cell Sub-populations. Circulation Research, 121(suppl_1), A478-A478.

Anti-LGR5-PE 130-111-201 DA04-10E9.9
Barker, N. et al. (2007) Identification of stem cells in small intestine and colon by marker gene Lgr5. Nature 449(7165): 1003-1007

Anti-CD45-APC 17-0451-82 30-F11
Cao Y, Trillo Tinoco J, Sierra R, Anadon C, Dai W, Mohamed E, et al. ER stress-induced mediator C/EBP homologous protein thwarts effector T cell activity in tumors through T-bet repression. Nat Commun. 2019;10:1280

Animals and other organisms

Policy information about studies involving animals; ARRIVE guidelines recommended for reporting animal research

Laboratory animals	Balb/c (stock #000651), C57BL/6J (called B6; stock #000664), B6.129(Cg)-Gt(ROSA)26Sortm4(ACTB-tdTomato,-EGFP)Luo/J (called R26mTmG/+; stock #007676), and B6.126S-Iduatm1.1Kmke/J (called Idua-W392X, stock #017681) mice were purchased from The Jackson Laboratory (Bar Harbor, ME). Both male and female animals were included. The age of breeding mice was between 3 and 6 months of age. The age of prenatally injected mice was embryonic day 15.5 at time of injection and 1-6 months for gross phenotypic analyses. The age of postnatally injected mice was 10 weeks of age. Control mice were between 1 and 6 months of age and age matched to experimental mice.
Wild animals	The study did not involve wild animals.
Field-collected samples	The study did not involve samples collected from the field.
Ethics oversight	The experimental protocols were approved by the Institutional Animal Care and Use Committee at CHOP and followed guidelines set forth in the National Institutes of Health's Guide for the Care and Use of Laboratory Animals.

Note that full information on the approval of the study protocol must also be provided in the manuscript.

Flow Cytometry

Plots

Confirm that:

☒ The axis labels state the marker and fluorochrome used (e.g. CD4-FITC).

☒ The axis scales are clearly visible. Include numbers along axes only for bottom left plot of group (a 'group' is an analysis of identical markers).

☒ All plots are contour plots with outliers or pseudocolor plots.

☒ A numerical value for number of cells or percentage (with statistics) is provided.

Methodology

Sample preparation	Cardiomyocytes, cardiac fibroblasts, and cardiac endothelial cells were isolated from in utero AAV.ABE.Idua injected Idua-W392X mice to assess for on-target gene editing in these cell populations. For cardiomyocyte isolation, freshly dissected hearts were transferred immediately to a petri dish containing ice cold calcium free Hanks Balanced Salt Solution (HBSS). A 10 mm2 section of LV was excised sharply and quartered. The Pierce Primary Cardiomyocyte Isolation Kit (Thermo Fisher Scientific) was used per the manufacturer's instructions except as follows. Enzyme supernatant and washings were reserved and combined to isolate non-cardiomyocyte cell fractions. After removal of supernatant, the remaining digestion products contained cardiomyocytes and were homogenized using 1mL pipette tips cut to 3-5mm diameters and then filtered through a 250µm tissue strainer to avoid shear damage. Serial gravity filtration on ice was then employed for up to 3 cycles of 20 minutes to enrich cardiomyocytes. Cardiomyocyte enrichment was verified via light microscopy with confirmation of a majority of sarcomere containing cells and visible contraction. Genomic DNA from the enriched cardiomyocyte population was isolated using the Quick Extract DNA Solution as per the manufacturer's instructions. The supernatants containing the non-cardiomyocyte cell fractions were subjected to flow cytometry sorting to isolate cardiac fibroblasts and endothelial cells (see below). For heart, after isolation of the enriched cardiomyocyte population, the remaining supernatant was centrifuged at 300 x g for 5 minutes. Pellets were resuspended in FACS staining buffer and then stained with anti-CD45-APC, anti-CD90.2-PE-Cyanine7, and anti-CD31-Brilliant Violet 421. BD FACS Aria (BD Biosciences, Franklin Lakes, NJ) was utilized to sort fibroblasts (CD45- CD90.2+), endothelial cells (CD45- CD31+), and bone marrow-derived cells (CD45+). For liver cells, freshly dissected livers were transferred immediately to a petri dish containing ice cold PBS, manually homogenized to create a single cell suspension, and resuspended in FACS staining buffer. Liver cells were subsequently stained with anti-CD45-APC and anti-LGR5-PE and then sorted into CD45+LGR5- (hematopoietic cells) and CD45-LGR5+ (liver progenitor cells).
Instrument	BD FACSAria
Software	BD FACSDiva v8.0.1
Cell population abundance	A minimum of 250,000 CD45-LGR5+ cells were obtained for each liver sample. After manual separation of cardiomyocytes and subsequent FACS sorting, approximately half of the sorted fraction was CD45-CD31+ and half was CD45-CD90.2+ on average. Similarly processed samples were backsorted to verify sample purity >90%.
Gating strategy	After plotting cells on SSC and FSC, approximately 80% cells were included in FACS samples to evaluate the non-debris population. Populations were further refined visually. CD45 antibody was used to gate the subsample for CD45- cells. Then, using isotype controls for each respective antibody with boundaries set at <0.1% to generate the most conservative estimates for "positive" samples. Cells were then sorted for the described antibodies.

☒ Tick this box to confirm that a figure exemplifying the gating strategy is provided in the Supplementary Information.
